# Role of Attitude, Body Image, Satisfaction and Socio-Demographic Variables in Cosmetic Surgeries of Iranian Students

**DOI:** 10.29252/wjps.9.2.186

**Published:** 2020-05

**Authors:** Parisa Kasmaei, Roghaye Farhadi Hassankiade, Mahmood Karimy, Sara Kazemi, Fatemeh Morsali, Shaghayegh Nasollahzadeh

**Affiliations:** 1 Research Center of Health and Environment, Department of Health Education and Promotion, School of Health, Guilan University of Medical Sciences, Rasht, Iran;; 2 Department of Biostatistics and Epidemiology, School of Public Health, Kermanshah University of Medical Sciences, Kermanshah, Iran;; 3 Social Determinants of Health Research Center, Saveh University of Medical Sciences, Saveh, Iran;; 4 Department of Health Education, Faculty of Medical Sciences, Tarbiat Modares University, Tehran, Iran;; 5 Expert of Family Health and Diseases, Asadabad University of Medical Sciences, Asadabad, Iran;; 6 School of Health, Guilan University of Medical Sciences, Rasht, Iran

**Keywords:** KEYWORDS

## Abstract

**BACKGROUND:**

Nowadays, cosmetic surgery is one of the most common types of surgeries all around the world including Iran and those demanding such surgeries are also growing in number. The present study is an attempt to determine the factors in the tendency towards cosmetic surgery in Iranian students.

**METHODS:**

In a descriptive-analytical study, all students at Guilan University of Medical Sciences related to health issues were enrolled. The participants (n=314) were selected through census sampling. Data was garnered using a multi-section questionnaire including socio-demographic variables, body image, body satisfaction, and cosmetic surgery intention. The questionnaires were completed as self-statement.

**RESULTS:**

The age range of the students was 18-55 years and the mean age was 25.07±7.41 years. Body mass index (BMI) of the participants was 23±3.82. Among socio-demographic variables, BMI, gender, family revenue, father’s job, marital status, mother’s job, and fathers’ literacy level were the predictors of intention for cosmetic surgery. In addition, among psychological variables, body satisfaction and image, and attitudes were predictors of intention for cosmetic surgery.

**CONCLUSION:**

Educational and psychological interventions are recommended to create body satisfaction, to develop positive attitudes toward one’s body, and to develop negative attitudes toward cosmetic surgery and the side effects. Apparently, providing an environment for physical activity and exercise, especially for girls would help the students in losing weight, remaining in shape and attenuating the tendency toward cosmetic surgery.

## INTRODUCTION

The desire for beauty is an age long comrade of the man so that man, as a being, likes beauty.^1^ Cosmetic surgery is of the latest man’s achievements to create beauty,^2^ and aims to alter body appearance for no medical reason or congenital/hereditary malformation and it can be effective in the improvement of quality of life.^3^ In general, cosmetic surgeries are not done due to medical urgency and beauty is the sheer reason for doing them.^4^ Today, one’s body form affects the way social relationships are formed and people try to build a non-questionable personality that fits day social values.^5^

As a result, the demand for cosmetic surgery is growing fast, so that 17.5 million people have undergone cosmetic surgery in USA in 2017, which is 137% higher than 2000.^6^ Consistent with the rest of the world, cosmetic surgeries have become very popular in Asian countries and among the youth in particular in recent years.^7^ For instance, eight out of every ten Korean women above 18 years old feel the need for cosmetic surgery.^8^ Reports show that Iran is one of the top countries in terms of the popularity of cosmetic surgeries and there is a growing demand for cosmetic surgeries in Iran.^9^

The increase in the rate of cosmetic surgery can be attributed to higher sensitivity of physical appearance in contemporary societies, lower cost of such interventions, and higher public awareness about cosmetic surgery for which the media is to be blamed. Other studies have highlighted individual factors like age, educational level, parents’ education, and social-economical status.^10^ With an increase in the awareness about cosmetic surgery, some authors have felt the urge to clarify the psychological factors in individuals who do cosmetic surgery. Previous studies have shown that there is a negative relationship between cosmetic surgery, body image and physical attractiveness.^11^

Moreover, factors like stress and anxiety, low self-esteem, body dissatisfaction, positive attitudes to cosmetic surgery, and depression are effective in cosmetic surgery.^12^^,^^13^ As the evidence shows, one reason for the increased popularity of cosmetic surgery is the positive attitudes towards it as a tool to improve one’s appearance.^14^ Attitudes are cognitive, emotional, and behavioral that determine whether an action is perceived as positive or negative.^15^ Positive attitudes towards cosmetic surgery are not the same in different cultures, so that they are a function of the general perception of people about cosmetic surgery.^16^

Body image is one of the psychological aspects with regard to cosmetic surgery. Body image includes thoughts, beliefs, evaluating emotions, and behaviors about the physical appearance of an individual.^17^ These are affected by several factors and might change in different situations. To improve their mental body image, people try different diets, exercises, or cosmetic surgeries.^18^ Dissatisfaction with physical appearance might convince individuals to adopt invasive methods (e.g. plastic surgery) to improve their appearance.^19^

Previous studies have shown that people with a negative image of their body have a higher tendency to cosmetic surgery.^19^^-^^21^ Farshidfar *et al.* showed that the fears and worries about physical appearance and body deformity were predictors of a tendency towards cosmetic surgery.^22^ The mental image of the body is one of the major concerns for the young and teenager. Individuals in this age range experience psychological and social changes.^23^ Such changes are more pronounced in students who experience considerable social pressures with regard to their physical appearance.^23^

Dissatisfaction with body image affects one’s psychological health and leads to physical and mental problems. A desire for beauty is a natural and common trait in man; however, it is stronger in individuals who are dissatisfied with their bodies. These individuals tend to become involved in unhealthy behaviors like food deprivation, using weight loss/gain drugs, and excessive exercises.^24^ Taking into account that the factors effective in the intention to do cosmetic surgery in a cultural background like Iranian cultural is different from other countries and that there is a high demand for cosmetic surgery in the youth,^25^ the present study was an attempt to determine the factors effective in the intention to do cosmetic surgery in Iranian university students. 

## MATERIALS AND METHODS

A cross-sectional descriptive-analytical study was conducted on a study population comprised of all students in health-related fields at Guilan University of Medical Sciences (North of Iran) in 2018. A sample group of 314 students was formed through the census sampling method. Inclusion criteria were current students in different health-related fields at the university, no psychological disorder, and desire to participate in the study. Data gathering tool was a multi-section standard questionnaire designed based on other studies. 

The questionnaire consisted of five sections including socio-demographical information (15 Items) such as age, gender, field of study, program, marital status, job, number of family members, birth order, parents’ education and job, average monthly revenue, ethnicity, weight and height, ideal weight and height, source of motivation and tendency to surgery, and the body part for cosmetic surgery. Section two of the questionnaire was based on Henderson-King’s attitude questionnaire.^26^

The questionnaire was comprised of 15 statements (e.g. “if cosmetic surgery could make someone happier with the way they look, then they should try it”). The questions of these sections were designed based on Likert’s seven-point score (completely agree=7 to completely disagree=1). The higher score of attitudes indicates a higher chance of undergoing cosmetic surgery. Henderson_King’s questionnaire has been validated in Iran*.*^10^ The reliability of the questionnaire was calculated here using Cronbach’s alpha equal to 0.92. 

Section three of the questionnaire was based on Littleton *et al.* body image questionnaire with 19 statements (e.g. “if cosmetic surgery has a positive effect on my future occupation, I would consider having it”).^27^ The statements of these sections were based on Likert’s five-point scale (always=5, mostly, sometimes, rarely, never=1). The higher score in this section meant a higher dissatisfaction with one’s body. This section was validated in Iran and Cronbach’s alpha of which was obtained equal to 0.90.^28^

The reliability of this part of the questionnaire was obtained equal to 0.88. Section 4 was consisted of 16 questions about body satisfaction. The statements were designed based on Likert’s seven-point scale (very satisfied=0, to very dissatisfied=7). This section of the questionnaire was developed and preliminarily validated by Slade *et al.*, and its reliability is supported in Iran (α=0.90). Here, Cronbach’s alpha of this section was obtained equal to 0.89. Section five of the questionnaire measured intention to do cosmetic surgery with one Yes/No question “Would you do cosmetic surgery in the future?”^29^

The study was approved by the Ethics Committee, Guilan University of Medical Sciences (IR.GUMS.REC.1395.385) in March 2017. Participation was voluntarily and the questionnaire was filled anonymously. In addition, the participants signed a letter of consent. The data were analyzed by SPSS software (Version 21, Chicago, IL, USA) using independent sample t-test (*p*=0.05) to compare the scores of attitudes, body satisfaction, and body image between the students with and without intention to do cosmetic surgery. 

A chi-squared test was used to find any relationship between the demographical variables and the intention and multiple logistic regression analysis was used to determine the factors effective in the intention to do cosmetic surgery. At first, univariate analysis was performed and only the independent variables that had a significant relationship with cosmetic surgery intention (*p*≤0.05) entered multiple logistic regression models. The independent variable in the study was “intention to do cosmetic surgery” and those with a positive attitude about the surgery (Yes) were encoded as 1 and those who were negative about the surgery (No) were encoded as 0. 

## RESULTS

The age range of the students was 18-45 years and the mean age was 25.07±7.41 years. Moreover, female participants constituted 84% of the students and mean body mass index (BMI) of the students was 23±3.82. The mean ideal BMI was 21.16±2.92 and 109 participants (34.7%) wanted to conduct cosmetic surgery in future. Among them, 22.9% wished nose surgery, 7.6% liked teeth cosmetic works, 3.8% asked for cheek and face surgery, 2.2% requested chin surgery, 1.9% demanded jaw surgery, and 3.6% searched for cosmetic surgery on the eyes and other parts of the body. 

Friends were the main source of intention for cosmetic surgery (48%) followed by the media (39%) and family (19%). A Chi-square test showed that among demographical variables, gender, marital status, fathers’ literacy and job, mother’s job, and students’ BMI had a significant relationship with intention to cosmetic surgery (*p*<0.05) (Table 1). The mean scores of attitudes, body image, and dissatisfaction in girls and unmarried individuals were higher than boys and married individuals. In addition, independent t-test showed a significant difference between the two groups in terms of the mean scores of attitudes, body image, and satisfaction (*p*<0.05). 

**Table 1:** Socio-demographics of participants according to cosmetic surgery intention

**Variable**		**Cosmetic surgery intention**		***p*** ** value**
		**Yes ** **N (%)**	**NO ** **N (%)**	
Sex	Female	101 (32.1)	163 (52)	0.002
	Male	8 (2.5)	42 (13.4)	
Marital status	Single	87 (27.7)	130 (41.4)	0.003
	Married	22 (7)	75 (23.8)	
Father’s job	Employee	51 (16)	90 (28.6)	0.005
	Worker/Farmer	7 (2.2)	44 (14)	
	Other	51 (16)	71 (22.6)	
Father’s literacy	Illiterate/ Elementary	9 (2.8)	55 (17.5)	0.001
	Middle school	16 (5)	19 (6)	
	High school/University	84 (26.7)	131 (41.7)	
Mother’s job	Housewife	78 (24.8)	177 (56.4)	0.005
	Employed	30 (9.5)	26 (8.3)	
Body mass index (BMI)	Underweight	17 (5.4)	13 (4.1)	0.02
	Normal	71 (22.6)	128 (40.7)	
	Over weight/Obese	24 (7.6)	60 (19)	

The ANOVA test showed a significant difference in the mean scores of attitudes, body image, and satisfaction based on BMI, father’s literacy, parents’ occupation status, and family revenue (*p*<0.05) (Table 2). To determine predictors of intention toward cosmetic surgery, multiple logistic regression was used and the results showed that among socio-demographic variables, BMI (OR=2.86; 95%, CI:1.37-5.95), gender (OR=2.79; 95% CI:1.25-6.23), family revenue (OR=2.69; 95% CI:1.20-6.01), father’s job (OR=2.41; 95% CI:1.06-5.45), marital status (OR=2.35; 95% CI:1.02-5.24), mother’s job (OR=1.99; 95% CI:1.03-3.87), and fathers’ literacy level (OR=1.09; 95% CI:1.01-1.18) were the predictors of intention toward cosmetic surgery. In addition, among psychological variables, body satisfaction (OR=3.72; 95% CI:1.32-10.43), body image (OR=2.91; 95% CI:1.29-6.57), and attitudes (OR=2.88; 95% CI:1.00-8.23) were predictors of intention for cosmetic surgery (Table 3). 

**Table 2:** Comparison of attitude, body image, and body satisfaction, according to socio-demographics variables

**Variable**		**Attitude **	**Body image **	**Body satisfaction**
		**Mean±SD**	**Mean±SD**	**Mean±SD**
Sex	Female	56.2±18.8	70.8**±**12.7	29.5**±**13.8
	Male	49.5±18.2	55.6**±**17.1	25.5**±**10.9
	*p* value	0.02	0.001	0.05
Marital status	Single	56.0±18.9	69.2±13.3	30.1±14.1
	Married	50.7±18.5	62.4±18.5	26.1±11.4
	*p* value	0.02	0.001	0.01
Father’s job	Employee	54.7±18.2	67.9±16.0	27.1±10.3
	Worker/Farmer	46.6±21.0	52.2±16.8	21.0±7.3
	Self-employed	59.7±21.3	70.5±13.6	28.5±9.4
	*p* value	0.003	0.001	0.001
Father’s literacy	Illiterate/ Elementary	47.8±16.9	60.3±19.6	24.2±8.5
	Middle school	51.9±19.6	63.6±16.8	28.9±13.5
	High school/University	56.8±18.4	68.1±14.7	32.1±14.3
	*p* value	0.001	0.006	0.02
Mother’s job	Housewife	45.2±13.6	65.1±15.0	25.3±10.5
	Employed	57.2±19.3	73.4±14.7	29.4±8.5
	*p* value	0.01	0.004	0.001
Body mass index (BMI)	Underweight	52.9±21.8	62.9±19.6	24.8±9.4
	Normal	58.1±20.3	64.8±17.4	30.2±11
	Over weight/Obese	61.3±19.8	70.6±16.1	33.4±14
	*p *value	0.02	0.04	0.001
Family revenue	Low	47.8±18.7	52.3±20.9	24.6±12.0
	Moderate	55.5±21.7	62.8±19.0	27.6±11.1
	High	60.3±21.3	69.4±15.0	30.3±10.9
	*p* value	0.001	0.001	0.003

**Table 3:** Uni-variate and multiple logistic regression model predicting intention toward cosmetic surgery

**Socio-demographic**	OR (95%CI)*	***p*** ** value**	OR (95%CI)**	***p*** ** value**
Sex				
Male	1.0 (Ref.)			
Female	3.61 (1.09-11.91)	0.03	2.79 (1.37-5.68)	0.005
Body mass index (BMI)				
Normal	1.0 (Ref.)			
Underweight	2.24 (0.86-5.84)	0.09	-	
Overweight/Obese	2.28 (1.32-3.94)	0.003	2.86(1.36-5.95)	0.005
Family revenue				
Low	1.0(Ref.)			
Moderate	1.45 (0.35-5.92)	0.60	-	
High	2.61 (1.44-4.71)		2.69 (1.20-6.01)	0.01
Father’s job				
Worker/Farmer	1.0 (Ref.)			
Employee	3.23 (1.0-10.12)	0.04	1.29 (0.78-2.12)	0.31
Self-employed	6.22 (1.78-21-68)	0.004	2.41 (1.06-5.45)	0.03
Marital status				
Married	1.0 (Ref.)			
Single	1.96 (1.12-3.41)		2.35 (1.02-5.24)	0.04
Mother’s job				
Housewife	1.0 (Ref.)			
Employed	3.21 (1.16-8.85)	0.02	1.99 (1.03-3.87)	0.04
Fathers education				
Illiterate/Elementary	1.0 (Ref.)			
Middle school	2.0 (0.43-9.70)	0.36	-	
High school/University	2.43 (1.20-4.93)	0.01	1.09 (1.01-1.18)	0.02
Body satisfaction	5.49 (2.46-12.25)	0.001	3.72 (1.32-10.43)	0.01
Body image	2.28 (1.32-3.94)	0.003	2.91 (1.29-6.57)	0.01
Attitudes	3.25 (1.46-7.20)	0.004	2.88 (1.00-8.23)	0.04

*Obtained from univariate analysis, **Adjusted OR obtained from multiple logistic regression analysis

## DISCUSSION

In this study, the effective factors in the intention toward cosmetic surgery in university students were studied. The results showed that the variables of body satisfaction and image were the two main effective factors in the intention of surgery. To explain this, social and cultural factors and an overemphasis on the physical appearance of individuals by society and media were notable. Today, people are bombarded with a large volume of advertisement about an ideal body and many have no chance to achieve such ideal. So that they develop negative body image and feel dissatisfied with their bodies. It was shown that the wider the gap between one’s image of the ideal body and the actual body, the higher the body dissatisfaction would be.^30^

The main areas of dissatisfaction has been discussed in women with their bodies, their appearance and weight.^31^ The volunteers of cosmetic and therapeutic surgery were compared and it was found that the body image in those who wanted cosmetic surgery was more negative than the other group.^32^ Others showed that a large number of individuals and most women wanted to change their body image.^33^ After the development of body dissatisfaction and negative self-image, it was demonstrated that people tried to change their physical image and completed the gap between the actual and ideal body image.^34^

The gravity of this variable can be explained by an analytical survey of the demographical variables of students as the students with a higher BMI had lower body satisfaction, more negative body image, and stronger desire to do cosmetic surgery. The findings showed that attitude was one of the effective and predictive variables for the intention to do cosmetic surgery that is consistent with previous studies on the students in Singapore.^12^^,^^16^ The growth of techniques to improve physical appearance has changed attitudes in people about cosmetic surgery, so that a larger group of the youth now has a more positive attitude about cosmetic surgery.^35^

A literature review showed that positive attitudes were positively related to acceptance of, approval of, and intention for cosmetic surgery. In addition, the attitudes are affected by several internal factors like body image and interpersonal factors, such as the effect of media, celebrities, and friends.^11^^,^^12^ In Iran, in the absence of formal fashion magazines and TV shows, students have limited access to fashion and beauty advertisements. This gives a stronger role to play to friends to form attitudes and tendencies to cosmetic surgery; which was supported by the findings.

As expected, gender was an effective factor in the tendency to cosmetic surgery and compared with men, women had more negative body image and lower body satisfaction. In addition, girls had a more positive attitude towards and intention to do cosmetic surgery. This is consistent with other researchers.^9^^,^^36^^,^^37^ According to the UK and USA associations of cosmetic plastic surgery, about 90% of all cosmetic surgeries in these two countries are done on women.^16^ It has been illustrated that despite the heavier investment in their bodies, women tended to have lower body satisfaction.^38^

This might be due to cultural-social pressure by the society on women, so that they feel the urge to be physically and sexually more attractive. In many societies, women are merely evaluated based on their physical attractiveness rather than their abilities and personal/social successes. Compared with men, women were noticed to experience more pressure to meet the social expectations about attractiveness, so that women are evaluated based on their bodies. Thus, women tended to be more inclined to cosmetic surgery.^16^

At any rate, cosmetic surgery is one of the tools that gives women the opportunity and capability to have a chance to resist against society. Women use cosmetic surgery in a way to gain power as they find power in beauty and attractiveness. Intention to do cosmetic surgery was also related to the father’s literacy level; so that the higher the father’s literacy, the stronger was the desire for cosmetic surgery. This can be explained by the fact that such surgeries are expensive and the higher income of more educated fathers facilitates doing such surgeries by students. This is consistent with a study in Saudi Arabia demonstrating that higher revenue was a factor in higher desire for cosmetic surgeries.^39^

Consistent with several researchers,^32^^,^^40^ unmarried participants had more desire for cosmetic surgery. This can be due to the prevalence of the beliefs in some unmarried individuals that physical beauty is a primary facilitator of marriage and to have a successful marriage, they needed to be as beautiful as possible. In terms of research limitations, it is notable that the study population was limited to young students and the findings cannot be generalized to all Iranian youth. 

Uncontrolled prevalence and popularity of cosmetic surgery is social damage that leads to several problems that affect not only the individual, but also the family and society. The study yielded a deep insight into the factors in cosmetic surgery by the youth in an Islamic society. The findings can be used for developing better programs and preventive interventions. Similar students on non-student youth and qualitative studies are recommended to achieve a deeper understanding of the factors effective in the intention of cosmetic surgery.
